# Antiobesity Effects of the Combined Plant Extracts Varying the Combination Ratio of* Phyllostachys pubescens* Leaf Extract and* Scutellaria baicalensis* Root Extract

**DOI:** 10.1155/2016/9735276

**Published:** 2016-03-30

**Authors:** Dong-Seon Kim, Seung-Hyung Kim, Jimin Cha

**Affiliations:** ^1^KM Convergence Research Division, Korea Institute of Oriental Medicine, 1672 Yuseong-daero, Yuseong-gu, Daejeon 305-811, Republic of Korea; ^2^Institute of Traditional Medicine and Bioscience, Daejeon University, Daejeon 300-716, Republic of Korea; ^3^Department of Microbiology, Faculty of Natural Science, Dankook University, Cheonan, Chungnam 330-714, Republic of Korea

## Abstract

The antiobesity effects of several different combinations of extracts (BS) prepared from two plants,* Phyllostachys pubescens* leaf (bamboo leaf: BL) and* Scutellaria baicalensis* root (SB), were investigated using a high fat diet (HFD) induced obese mouse model. In order to find the most effective mixture among the mixtures of the two plant extracts, experimental preparations were made by combining BL and SB by different proportions of 3 : 1 (BS31), 2 : 1 (BS21), 1 : 1 (BS11), 1 : 2 (BS12), and 1 : 3 (BS13). Body weight, weight of adipose tissues, size of adipocytes, levels of glucose, leptin and adiponectin, and lipid profile in serum, and fat accumulation in liver were investigated. We have found that BS21 is the most effective in antiobesity among the five mixtures investigated, indicated by reduction in body weight gain, total mass of adipose tissue, and the size of adipocyte. In addition, BS21 has shown to be beneficial in serum lipid profile, levels of glucose, leptin, and adiponectin in serum, and fat accumulation in liver. By chromatographic separation of BS21, the two maker compounds, isoorientin and baicalin, were identified and quantified for the standardization of BS21.

## 1. Introduction

Obesity is a medical condition in which excess body fat has accumulated to the extent that it may cause negative effects on health leading to reduced life expectancy and/or increased serious health problems. It has been increasingly believed that obesity is associated with numerous metabolic disorders, including hyperlipidemia and type 2 diabetes mellitus [1], cardiovascular diseases [2], such as hypertension and atherosclerosis [3], and many other disorders, such as osteoarthritis [4] and certain types of cancer [5]; all of these conditions can seriously increase morbidity and mortality [6]. It has been also recognized that obesity is correlated with psychological functioning [7]. Pharmacological therapies to treat obesity have been reviewed, classified into four categories: fat blockers, antidepressants, stimulants, and diabetes medications [8]. New therapeutic approaches for the treatment of obesity have been proposed, focusing on the control of energy balance [9].

Obesity has a multifactorial nature resulting from genetic, physiological, sociocultural, psychological, and environmental factors that lead to an energy imbalance [10]. It has been recognized that no one medication is effective in every patient with obesity, and the ideal medication has to be accompanied by lifestyle changes, dietary modification, and increased physical activity in order to treat obesity effectively [10].

With an increasing prevalence of being overweight or obesity in all ages, herbal usage to achieve weight loss has become a major focus for improving public health in many countries. Bangpoongtongsungsan (BPT), a traditional herbal medicine composed of 18 crude medicinal herbs, has been used as an antiobesity treatment in overweight patients [11]. In mice fed with a high fat diet (HFD), BPT appeared to decrease the weight of white adipose tissue and the size of adipocytes [12]. It is of note that these medicinal herbs have beneficial effects in obesity without significant side effects, suggesting that these herbs can offer an excellent alternative strategy to develop safe and effective antiobesity drugs [13, 14].

According to the traditional medicine in Korea and China, bamboo leaves have been used to treat palsy and hypertension [15, 16]. Antioxidant and anticoagulant effects of* Phyllostachys pubescens *leaf have been reported [15]. Isoorientin, one of the flavonoids found in* Phyllostachys pubescens *leaf, has been reported to inhibit adipogenesis in 3T3-L1 cells [17].* Scutellaria baicalensis* root has been traditionally used for diuretic, antidiarrhea, and anti-inflammation effects and recently reported to reduce the food intake, to improve serum lipid profile, and to increase the total antioxidant status in serum [18]. A recent study also suggested that* Scutellaria baicalensis* extract could be used as potent therapeutic agents for the treatment of weight gain and hypertriglyceridemia [19].

In our previous study, we selected several plants to screen for antiobesity effects among the plants known as safe to use for dietary purpose.* Phyllostachys pubescens* leaf (bamboo leaf: BL) and* Scutellaria baicalensis* root (SB) showed the most reliable antiobesity effects among the plants that we investigated. We found that the 1 : 1 (w/w) mixture of the two plants extracts demonstrated synergistic antiobesity effects [20]. In this study, after exploring the antiobesity effects of the two plants, we investigated whether the different combinations of the two plants extracts' mixtures have effects on antiobesity or not. This study was also designed to estimate to what extent the mixtures of different ratios show potency enabling us to develop an antiobesity agent and to find out the most effective mixture and, therefore, to standardize the most effective mixture for commercialization.

## 2. Materials and Methods

### 2.1. Preparation of BL Extract and SB Extract and Various Mixtures in Different Ratios of Each Extract

*Phyllostachys pubescens *leaf was collected in Damyang, Korea.* Scutellaria baicalensis* root was purchased as a dried herb from Omniherb Co., Yeoungcheon, Korea, and was authenticated by the Classification and Identification Committee of KIOM (Korea Institute of Oriental Medicine) based upon its microscopic and macroscopic characteristics. The committee was composed of nine experts working in the fields of plant taxonomy, botany, pharmacognosy, or herbology. The voucher specimens (BL-20120727; SB-20120914) were deposited at the herbarium of KIOM.

The same extraction procedure was applied for both plants. 1 kg of the dried plant material was extracted twice with 80% ethanol (v/v) in water at 82°C for 3 hours. The extract was filtered and then evaporated under the reduced pressure in a rotary evaporator. The yields of BL extract and SB extract were 89.1 g and 193 g, respectively. The five BS mixtures were prepared by mixing BL extract and SB extract at the weight ratios of 3 : 1, 2 : 1, 1 : 1, 1 : 2, and 1 : 3 to give BS31, BS21, BS11, BS12, and BS13, respectively.

### 2.2. Animals and Experimental Diets

Male C57B1/6 mice were purchased from Daehan Biolink Co., Eumsung, Korea, and maintained for 2 weeks with sufficient supply of commercial diet (AIN-76A diet, Ralston Purina, St. Louis, MO, USA) and water prior to the experiments. 10-week-old mice were housed in the air-conditioned SPF animal room having a 12 h light/12 h dark cycle at 25 ± 2°C temperature and 50 ± 5% humidity. They were allowed to have access to the laboratory diet and water ad libitum. All experimental protocols were conducted according to the guidelines of NIH (National Institutes of Health) and were approved beforehand by the Animal Care Committee of KIOM.

To induce obesity, the mice were fed with the high fat diet (HFD: Rodent Diet D12492, Research Diets, New Brunswick, NJ, USA) consisting of 60% fat, 20% protein, and 20% carbohydrate as was described in the previously published report [21]. The normal diet group was fed with the standard chow diet (Orient Bio Inc., Seongnam, Korea) commercially available. HCA (Garcinia Cambogia) [22] and XNC (Xenical) [23] were used as positive control groups since they were well known and available in public as antiobesity agents. After 2 weeks of adaptation period, the experiments were initiated when the weights of mice reached 28-29 g by feeding with the high fat diet. The mice were then fed for 6 weeks with group specific diets. The mice were randomly divided into nine groups (*n* = 7) and separately fed with the normal diet (ND), the high fat diet (HFD), HFD plus HCA (HCA), HFD plus XNC (XNC), HFD plus BS31 (BS31), HFD plus BS21 (BS21), HFD plus BS11 (BS11), HFD plus BS12 (BS12), and HFD plus BS13 (BS13). 100 mg/kg/day of oral dosage was applied for all the mice in the experimental groups except XNC group, the dosage of which was chosen to be 15.6 mg/kg/day with reference to the dosage range used in the previous report [24]. All the preparations were made by suspending in normal saline and administered orally by using mouse Zonde. ND and HFD control groups were treated with vehicle (normal saline) only.

### 2.3. Measurement of Body Weight Gain and Food Intake

Body weight gain and the amount of food intake were measured at the same time and the same day of a week during 6 weeks of experimental period. Average body weight gain and average amount of food intake were daily calculated and recorded. FER (food efficiency ratio) was calculated by (total weight gain/total food intake) × 100.

### 2.4. Serum Assays for Biochemical Parameters

At the end of 6-week experimental period, the mice were fasted for 15 hours prior to sacrifice. Blood samples were centrifuged at 3000 rpm for 15 min at 4°C. The separated serum samples were stored at −70°C. The serum levels of triglyceride, total cholesterol, high density lipoprotein cholesterol (HDL), low density lipoprotein cholesterol (LDL), glucose, alanine aminotransferase (ALT), aspartate aminotransferase (AST), and creatinine were analyzed by automatic biochemical analyzer (Hitachi-720, Hitachi Medical, Japan). The serum concentrations of leptin and adiponectin were assayed with mouse ELISA (enzyme-linked immunosorbent assay) kits (R&D Systems, Minneapolis, MN, USA).

### 2.5. Measurement of Adipose Tissue Weight and Histological Observation

After the blood collection, liver, kidney, spleen, and inguinal, epididymal, and perirenal adipose tissues were removed from the mice and weighed immediately. For histochemistry, the tissues were fixed in 10% neutral formalin solution for one day and embedded in paraffin. All tissues were sliced to 6 *µ*m in thickness and stained with H&E (hematoxylin and eosin). To measure the size of adipocytes, the area comprising 20 adipocytes in stained sections was measured by light microscope (Olympus BX51, Olympus Optical Co., Japan) with the aid of image analysis program (Image-Pro Plus 5.0, Media Cybernetics, Silver Spring, MD, USA). Histological analysis was performed using the samples of the collected tissues prepared.

### 2.6. High Performance Liquid Chromatography Analysis for Identifying the Marker Compounds of BS21

HPLC-grade reagents, acetonitrile, and water were obtained from J. T. Baker (Phillipsburg, NJ, USA). All the other chemicals used in this work were of a reagent grade. The samples were analyzed by reverse phase-high performance liquid chromatography of Waters Alliance 2695 system (Waters Co., Milford, MA, USA) coupled with 2996 photodiode array detector. Phenomenex Luna C18 column (250 mm × 4.6 mm × 5 *µ*m, Phenomenex, Torrance, CA, USA) was used for the stationary phase and the mobile phase was composed of 0.1% (v/v) trifluoroacetic aqueous solution (A) and acetonitrile (B). At zero time, the mobile phase consisted of 90% A and 10% B and was held for 10 min. From 10 to 40 min a gradient was applied to 55% A and 35% B, which was followed by a wash with 100% B for 10 min and a 15 min equilibration period at 90% A and 10% B. For the separation, 1.0 mL/min of flow rate and 20 *µ*L of injection volume were kept throughout the analysis that was performed at 40°C.

Identification of the constituents of BS21 was made by comparing retention times and UV spectra for the peaks of HPLC/PDA chromatogram to those of commercially available standards. For each compound, peak area was determined at 350 nm. The calibration curve of the standards ranging from 6.25 to 200 *µ*g/mL (6 levels) for isoorientin and from 12.5 to 400 *µ*g/mL (6 levels) for baicalin revealed a good linearity.

Quantitation of the marker compounds of BS21 was made in comparison to the mixture of external standards of known concentration. Quantitative measurements were made in duplicate before and after the batch samples. The peak areas were used to calculate the contents of the compounds in the samples.

### 2.7. Statistical Analysis

Differences between groups were assessed by an analysis of variance (ANOVA) followed by Duncan's multiple-range test. All data are presented as mean ± SEM (Standard Error of the Mean). Differences were considered significant when the *p* values were less than 0.05.

## 3. Results

### 3.1. Effects of BS Mixtures on Body Weight, Food Intake, and Food Efficiency Ratio

**Figure 1:**
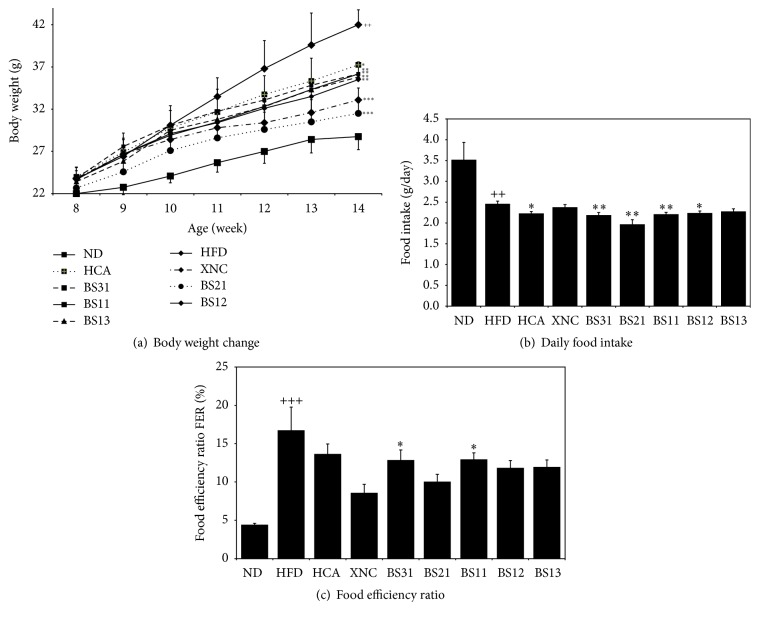
Effects of BS mixtures on (a) body weight gain, (b) food intake, and (c) food efficiency ratio in mice consuming high fat diet. The food efficiency ratio is calculated by (daily body weight gain/daily food intake) × 100. ND: normal diet, HFD: high fat diet control, HCA: high fat diet plus 100 mg/kg/day of Garcinia Cambogia, XNC: high fat diet plus 15.6 mg/kg/day of Xenical, BS31: high fat diet plus 100 mg/kg/day of BS31, BS21: high fat diet plus 100 mg/kg/day of BS21, BS11: high fat diet plus 100 mg/kg/day of BS11, BS12: high fat diet plus 100 mg/kg/day of BS12, and BS13: high fat diet plus 100 mg/kg/day of BS13. Values are expressed as mean ± SEM (*n* = 7). ^++^ *p* < 0.01 and ^+++^ *p* < 0.001 (compared with ND) and ^*∗*^ *p* < 0.05, ^*∗∗*^ *p* < 0.01, and ^*∗∗∗*^ *p* < 0.001 (compared with HFD) express significant differences as determined by Duncan's multiple-range test.

HFD control group gained significantly more weight compared to ND group, and the positive control groups (HCA and XNC) and BS (mixture of BL extract and SB extract) treated groups gained significantly less weight compared to HFD (Figure 1(a)). Although average daily body weight gain was considerably reduced in all positive control groups and BS treated groups compared to HFD (Table 1), only Xenical (XNC) and BS21 demonstrated statistically significant reduction among them (*p* < 0.01 for XNC and *p* < 0.001 for BS21).

Daily food intake was significantly decreased in HFD control group compared to ND group. However there was only a slight change in daily food intake between HFD control group and the positive control groups or BS treated groups (Figure 1(b)). Food efficiency ratio was significantly increased in HFD control group compared to ND group. BS31 and BS11 groups showed statistically significant reduction in food efficiency ratio compared to HFD control group (Figure 1(c)).

### 3.2. Effects of BS Mixtures on Serum Lipid Profile

**Figure 2:**
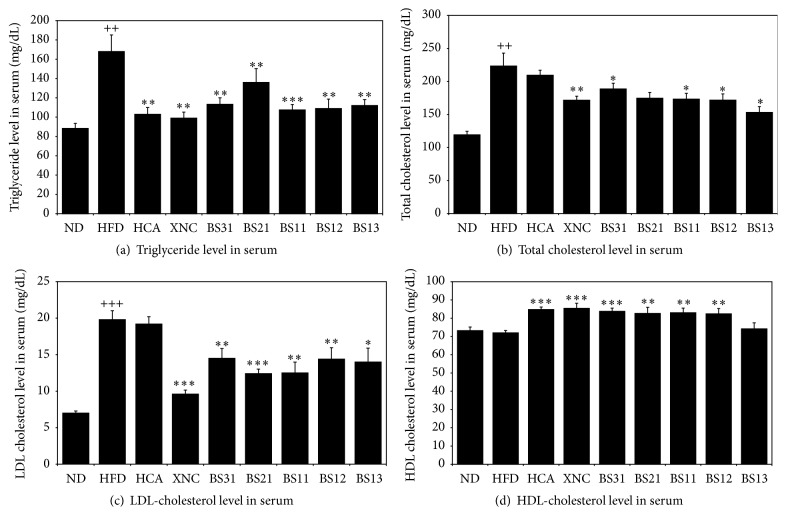
Effects of BS mixtures on the levels of (a) triglyceride, (b) total cholesterol, (c) LDL-cholesterol, and (d) HDL-cholesterol in serum. Values are expressed as mean ± SEM (*n* = 7). ^++^ *p* < 0.01 and ^+++^ *p* < 0.001 (compared with ND) and ^*∗*^ *p* < 0.05, ^*∗∗*^ *p* < 0.01, and ^*∗∗∗*^ *p* < 0.001 (compared with HFD) express significant differences as determined by Duncan's multiple-range test.

HFD control group showed significant increases in all parameters of the serum lipid profile compared to ND group except HDL-cholesterol (Figure 2). Compared to HFD control group, almost all BS treated groups showed to decrease significantly the levels of triglycerides, total cholesterol, and LDL-cholesterol in serum (Figures 2(a), 2(b), and 2(c)) and to increase significantly HDL-cholesterol level in serum (Figure 2(d)). HCA group showed little effects on the levels of total cholesterol and LDL-cholesterol in serum, while BS groups significantly reduced.

### 3.3. Effects of BS Mixtures on Energy Balancing Metabolism

**Figure 3:**
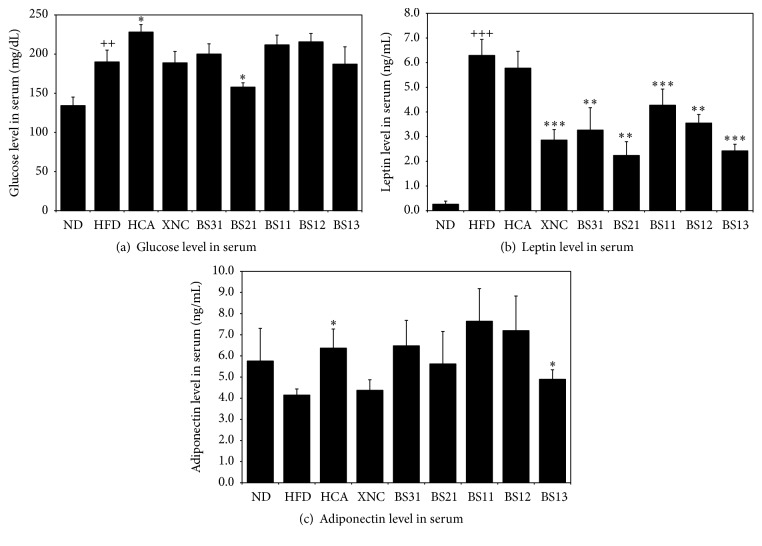
Effects of BS mixtures on the levels of (a) glucose, (b) leptin, and (c) adiponectin in serum. Values are expressed as mean ± SEM (*n* = 7). ^++^ *p* < 0.01 and ^+++^ *p* < 0.001 (compared with ND) and ^*∗*^ *p* < 0.05, ^*∗∗*^ *p* < 0.01, and ^*∗∗∗*^ *p* < 0.001 (compared with HFD) express significant differences as determined by Duncan's multiple-range test.

Serum glucose level was increased significantly in HFD control group compared to ND group. Only BS21 among BS treated groups showed statistically significant reduction in serum glucose level compared to HFD control group (Figure 3(a)). Serum leptin levels were increased significantly in HFD control group compared to ND group and significantly decreased in BS groups compared to HFD control group (Figure 3(b)). BS treated groups seemed to have a tendency to upregulate serum adiponectin levels (Figure 3(c)). Among the five BS mixtures, BS21 appeared to have better effects on energy balancing metabolisms compared to the other BS groups (Figure 3).

### 3.4. Effects of BS Mixtures on Serum Toxicity Markers

**Figure 4:**
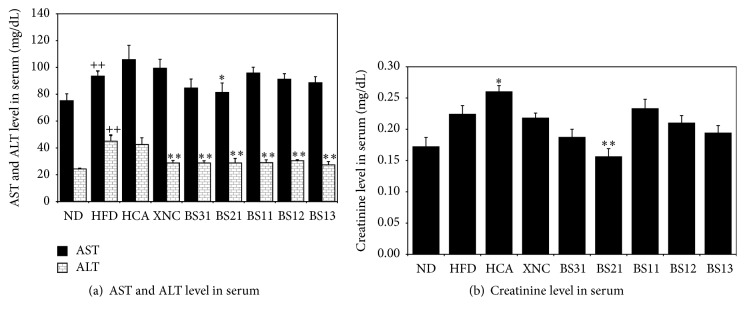
Effects of BS mixtures on the levels of ALT, AST, and creatinine in serum. Values are expressed as mean ± SEM (*n* = 7). ^++^ *p* < 0.01 (compared with ND) and ^*∗*^ *p* < 0.05 and ^*∗∗*^ *p* < 0.01 (compared with HFD) express significant differences as determined by Duncan's multiple-range test.

To evaluate both potential toxicity and protective effects of BS mixtures, serum toxicity markers for liver (ALT, AST) and kidney (creatinine) were assayed at the end of the experimental period. The levels of ALT and AST in serum were significantly increased in HFD control group compared to ND group (Figure 4(a)). Serum ALT level was significantly decreased in all BS groups while serum AST level was significantly decreased only in BS21 group (Figure 4(a)). There was no significant decrease in serum creatinine level in BS groups except BS21 group, which showed significant decrease in serum creatinine level (Figure 4(b)). On the whole, BS21 treatment seemed to cause no detectable adverse toxic effects and to protect to some extent the livers and kidneys of mice.

### 3.5. Effects of BS Mixtures on Fat Deposit (Weight of Adipose Tissues and Size of Adipocyte) and Histological Observations

**Figure 5:**
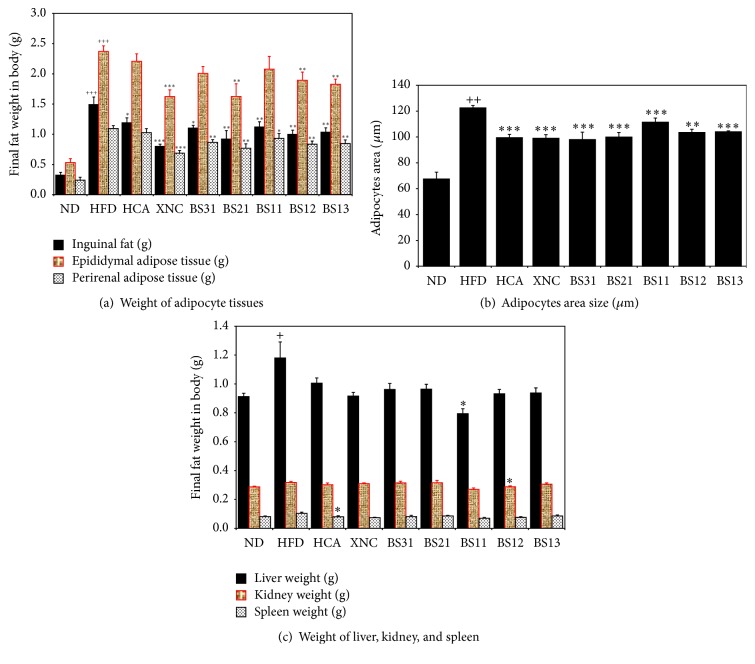
Effects of BS mixtures on fat deposit: (a) the weight of inguinal, epididymal, and perirenal adipose tissues, (b) adipocyte area size, and (c) the weight of liver, kidney, and spleen. Values are expressed as mean ± SEM (*n* = 7). ^+^ *p* < 0.05, ^++^ *p* < 0.01, and ^+++^ *p* < 0.001 (compared with ND) and ^*∗*^ *p* < 0.05, ^*∗∗*^ *p* < 0.01, and ^*∗∗∗*^ *p* < 0.001 (compared with HFD) express significant differences as determined by Duncan's multiple-range test.

The weights of inguinal and epididymal adipose tissues were increased significantly in HFD control group compared to ND group (Figure 5(a)). The weights of inguinal and perirenal adipose tissues were decreased significantly in all BS groups compared to HFD control group.

Also BS21, BS12, and BS13 showed to decrease significantly the weight of epididymal adipose tissue compared to HFD control group. XNC group and BS21 group showed the most effective and statistically significant decreases (*p* < 0.001 for XNC and *p* < 0.01 for BS21) in the weights of inguinal, epididymal, and perirenal adipose tissues compared to HFD control (Figure 5(a)) while HCA group showed a little effect on reduction of fat deposit. The outcome of the other BS groups (BS31, BS11, BS12, and BS13) lay in between those of XNG/BS21 groups and HCA group.

**Figure 6:**
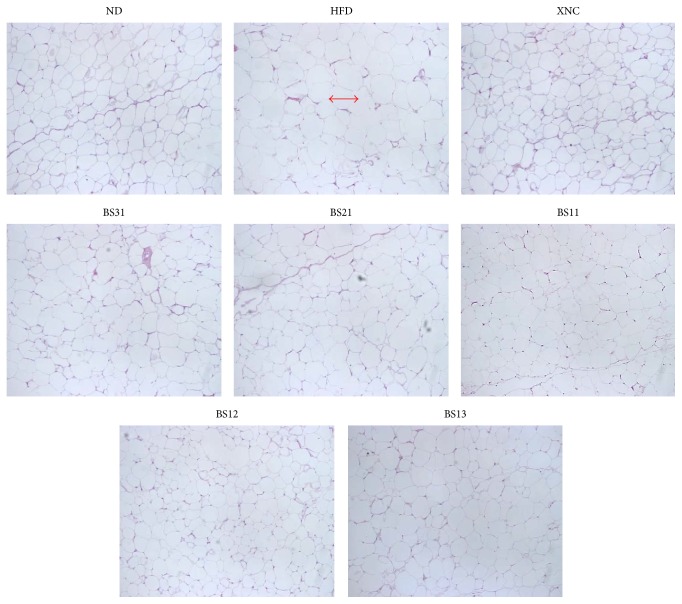
Effects of BS mixtures on adipocyte size. Histological observations of H&E stained adipose tissues of the experimental groups. Representative photographs of H&E stained epididymal adipose tissue (magnification: 400x). The arrow for the control group indicates the size of an adipocyte.

To estimate the adipocyte size, the area comprising 20 adipocytes in H&E stained sections was measured. HFD control group showed significant increase in adipocyte area size compared to ND group (Figure 5(b)). All the positive control groups and BS groups showed significant decreases in adipocyte area size compared to HFD control group (Figure 5(b)). These results were supported by the histological observations that demonstrated clearly the difference in adipocyte size among the experimental groups (Figure 6). We presume in the consideration of these results that the decrease in body fat mass by BS treatment is partly due to the decrease in adipocyte size. We obtained no statistically significant results with respect to the weight of liver, kidney, and spleen (Figure 5(c)). The weight of liver appeared to show a tendency to be decreased in BS groups compared to HFD control group. However, BS groups showed no significant reduction in liver weight increment except BS11, which showed significant reduction (Figure 5(c)).

**Figure 7:**
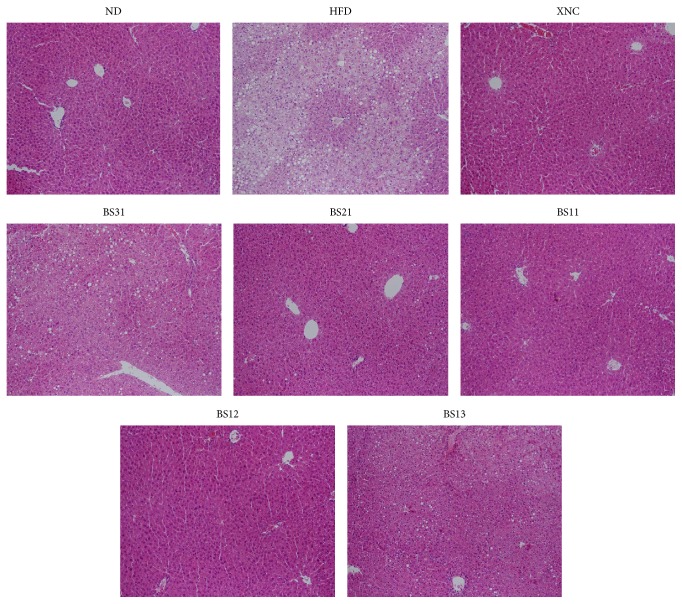
Histological profile of the representative H&E stained liver tissue section for the experimental groups (magnification, 200x).

Our data from histochemistry showed that the liver of HFD control group showed more extensive lipid droplet accumulation compared to ND group in histological observations (Figure 7). The liver of HFD control group contained macrovesicular lipid droplets as well as numerous microlipid droplets demonstrating a typical fatty liver developed by high fat diet. BS groups showed less lipid droplet accumulation than HFD control group (Figure 7). In particular, the liver conditions of BS21 and BS12 groups appeared to be close to those of ND group.

### 3.6. Chromatographic Separation of BS21 to Identify Marker Compounds

**Figure 8:**
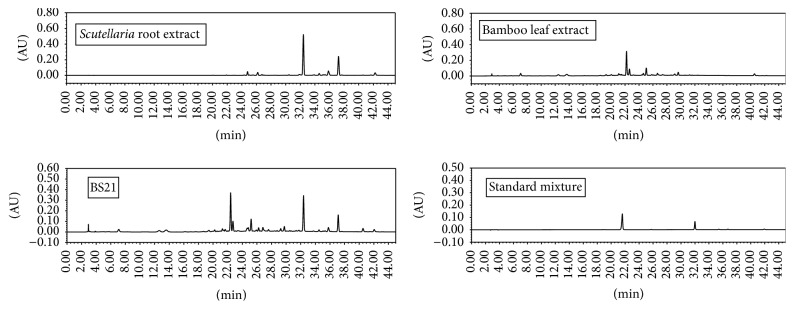
HPLC chromatograms of 80% (v/v) ethanol extracts of* Scutellaria* root and bamboo leaf, the mixture of two extracts (BS21), and the mixture of two reference standards, isoorientin (1) and baicalin (2). UV peaks were detected at 350 nm. Isoorientin and baicalin appeared at the retention times of approximately 22.4 min and 32.1 min, respectively.

HPLC/PDA chromatograms of BS21,* Scutellaria* root extract, and bamboo leaf extract are shown in Figure 8. The two marker compounds of BS21 were determined from the major peaks of HPLC/PDA chromatogram in comparison to the retention times and UV spectra of commercially available standards. As shown in Figure 8, the high performance liquid chromatographic analysis of BS21 revealed two major compounds, isoorientin originated from bamboo leaf extract and baicalin originated from* Scutellaria* root extract at the retention times of approximately 22.4 min and 32.1 min, respectively. Quantitation of the two marker compounds, isoorientin and baicalin, was made by chromatographic comparison between BS21 and mixture of the two commercial standards of isoorientin and baicalin (Figure 8). The result revealed that BS21 contained 7.2 ± 0.5 mg/g of isoorientin and 64.7 ± 3.2 mg/g of baicalin.

## 4. Discussion

In this study, we found that BS mixtures prepared from mixing with different ratios of the two herbal extracts,* Phyllostachys pubescens* leaf and* Scutellaria baicalensis* root, decreased body weight gain of mice fed with a high fat diet. BS21 treatment appeared to be the most effective in decreasing body weight gain among the five BS mixtures. The inhibitory effect of BS21 on the body weight gain was similar to that of Xenical, a well known prescription drug. The data from the measurement of daily amount of food intake and food efficiency ratio also suggested that BS21 was the most effective in reduction of body weight gain among the five BS mixtures.

The treatment of BS mixtures showed significant reductions in the amount of inguinal, epididymal, and perirenal adipose tissues. In the estimation of adipocyte size by measuring the area comprising 20 adipocytes, BS groups showed significant decreases in adipocyte size. This result was supported by comparative microscopic observations, between BS groups and HFD control group, made on the adipocytes of the stained adipose tissue slices. BS21 treatment appeared to be most effective for the inhibition of fat accumulation and adipocyte size expansion in adipose tissues.

Considering the results of body weight gain and internal fat mass, we made an assumption that BS21 treatment had potency to tackle obesity and its associated disorders.

HFD control group showed to increase significantly the levels of triglyceride, total cholesterol, and LDL-cholesterol in serum compared to ND group, demonstrating the development of hyperlipidemia (hypercholesterolemia and hypertriglyceridemia). BS groups showed to decrease significantly the levels of triglycerides, total cholesterol, and LDL-cholesterol in serum and to increase significantly HDL-cholesterol level in serum compared to HFD control group. The result suggested a possibility for the use of BS to help to prevent and/or relieve from adverse events caused by hyperlipidemia.

The increment of liver weight most probably by fat deposition seemed to reduce in BS groups compared to HFD control. In histological observations, the liver of HFD control group contained macrovesicular lipid droplets as well as numerous microlipid droplets, demonstrating a typical fatty liver developed by a high fat diet. BS groups showed less accumulation of lipid droplet in liver compared to HFD control group. We suggest in consideration of this result that BS treatment may help to prevent and/or relieve from fatty liver.

Adiponectin was known to modulate a number of metabolic processes, including glucose regulation and fatty acid oxidation [25]. Adiponectin was also reported to be inversely correlated with body fat percentage in adults and to mediate insulin-sensitizing effect to ameliorate hyperglycemia and hyperinsulinemia without inducing weight gain or even inducing weight loss [26]. The reduction of adiponectin level in serum was associated with insulin resistance, dyslipidemia, and atherosclerosis [27]. In obesity, a decreased sensitivity to leptin occurred, resulting in an inability to detect satiety despite the accumulation of high energy [28]. BS21 showed to lower the levels of glucose and leptin in serum and to elevate adiponectin level in serum.

Therefore, BS21 seemed to influence insulin sensitizing, fat mass reduction, and weight loss with the aids of numerous energy related processes mediated by reduced serum level of leptin and elevated serum level of adiponectin in obese conditions.

In the evaluation of the levels of ALT, AST, and creatinine in serum, BS treatment appeared to cause no detectable adverse toxic effects and to protect to some extent the livers and kidneys.

For the standardization of BS21, we identified two marker compounds, isoorientin and baicalin, each of which is the highest content constituent of* Phyllostachys pubescens* leaf extract and* Scutellaria baicalensis* root extract, respectively. Both baicalin [29] and isoorientin [17] have been reported to work as antiadipogenic regulators of the adipogenesis pathway.

## 5. Conclusions

In this study, BS21 showed the most reliable antiobesity effects among the five BS mixtures.

We demonstrated that BS21 treatment significantly lowered body weight gain. This study also showed that BS21 treatment effectively reduced adipose tissue mass as well as adipocyte size and improved positively the serum lipid profile including triglycerides, total cholesterol, LDL-cholesterol, and HDL-cholesterol.

BS21 treatment showed remarkable reduction in lipid droplet accumulation in fatty liver induced by a high fat diet and reduction in the serum glucose level. BS21 treatment also lowered the serum leptin level and elevated serum adiponectin level.

We, therefore, suggest as an extension of this study to explore further the possibilities of BS21 to apply for preventing and/or relieving from obesity and from hyperlipidemia, fatty liver, and other adverse events that may occur concomitantly with obesity.

By chromatographic separation of BS21, the two maker compounds, isoorientin for* Phyllostachys pubescens* leaf extract and baicalin for* Scutellaria baicalensis* root extract, were identified and quantified for the standardization of BS21. The two compounds, isoorientin and baicalin, were of extraordinarily high content in* Phyllostachys pubescens* leaf extract and* Scutellaria baicalensis* root extract, respectively. Therefore, isoorientin and baicalin were chosen as marker compounds considering profitability as quality control markers in comparison to the other constituents in trace level.

## Figures and Tables

**Table 1:** Effects of BS mixtures on average daily body weight gain in HFD induced obese mice.

	ND	HFD	HCA	XNC	BS31	BS21	BS11	BS12	BS13
Body weight gain (g/day)	0.153 ± 0.008	0.410 ± 0.075^++^	0.301 ± 0.03	0.202 ± 0.03^*∗∗*^	0.280 ± 0.02	0.200 ± 0.02^**∗∗∗**^	0.284 ± 0.02	0.260 ± 0.022	0.270 ± 0.022

ND: normal diet, HFD: high fat diet control, HCA: high fat diet plus 100 mg/kg/day of Garcinia Cambogia, XNC: high fat diet plus 15.6 mg/kg/day of Xenical, BS31: high fat diet plus 100 mg/kg/day of BS31, BS21: high fat diet plus 100 mg/kg/day of BS21, BS11: high fat diet plus 100 mg/kg/day of BS11, BS12: high fat diet plus 100 mg/kg/day of BS12, and BS13: high fat diet plus 100 mg/kg/day of BS13.

Values are expressed as mean ± SEM (*n* = 7). ^++^ *p* < 0.01 (compared with ND) and ^*∗∗*^ *p* < 0.01 and ^*∗∗∗*^ *p* < 0.001 (compared with HFD) express significant differences as determined by Duncan's multiple-range test.
